# Identification of a serum biomarker panel for the differential diagnosis of cholangiocarcinoma and primary sclerosing cholangitis

**DOI:** 10.18632/oncotarget.24732

**Published:** 2018-04-03

**Authors:** Joy Cuenco, Natascha Wehnert, Oleg Blyuss, Anna Kazarian, Harry J. Whitwell, Usha Menon, Anne Dawnay, Michael P. Manns, Stephen P. Pereira, John F. Timms

**Affiliations:** ^1^ Institute for Women's Health, University College London, London, WC1E 6BT, UK; ^2^ Hannover Medical School, Department of Gastroenterology, Hepatology and Endocrinology, Hannover, 30625, Germany; ^3^ Clinical Biochemistry, University College London Hospitals NHS Foundation Trust, London, W1T 4EU, UK; ^4^ Institute for Liver and Digestive Health, University College London, Royal Free Hospital, London, NW3 2PG, UK

**Keywords:** cholangiocarcinoma, biliary tract cancer, serum biomarker, primary sclerosing cholangitis, differential diagnosis

## Abstract

The non-invasive differentiation of malignant and benign biliary disease is a clinical challenge. Carbohydrate antigen 19-9 (CA19-9), leucine-rich α2-glycoprotein (LRG1), interleukin 6 (IL6), pyruvate kinase M2 (PKM2), cytokeratin 19 fragment (CYFRA21.1) and mucin 5AC (MUC5AC) have reported utility for differentiating cholangiocarcinoma (CCA) from benign biliary disease. Herein, serum levels of these markers were tested in 66 cases of CCA and 62 cases of primary sclerosing cholangitis (PSC) and compared with markers of liver function and inflammation. Markers panels were assessed for their ability to discriminate malignant and benign disease. Several of the markers were also assessed in pre-diagnosis biliary tract cancer (BTC) samples with performances evaluated at different times prior to diagnosis. We show that LRG1 and IL6 were unable to accurately distinguish CCA from PSC, whereas CA19-9, PKM2, CYFRA21.1 and MUC5AC were significantly elevated in malignancy. Area under the receiver operating characteristic curves for these individual markers ranged from 0.73–0.84, with the best single marker (PKM2) providing 61% sensitivity at 90% specificity. A panel combining PKM2, CYFRA21.1 and MUC5AC gave 76% sensitivity at 90% specificity, which increased to 82% sensitivity by adding gamma-glutamyltransferase (GGT). In the pre-diagnosis setting, LRG1, IL6 and PKM2 were poor predictors of BTC, whilst CA19-9 and C-reactive protein were elevated up to 2 years before diagnosis. In conclusion, LRG1, IL6 and PKM2 were not useful for early detection of BTC, whilst a model combining PKM2, CYFRA21.1, MUC5AC and GGT was beneficial in differentiating malignant from benign biliary disease, warranting validation in a prospective trial.

## INTRODUCTION

Biliary tract cancer (BTC) comprises tumours of the gallbladder and bile ducts, the commonest form of which is cholangiocarcinoma (CCA). CCA cells tend to infiltrate and spread along the biliary tract such that patients often have minimal clinical symptoms and present late, usually with cholestasis and evidence of locally advanced or metastatic disease on imaging. Consequently, five-year survival rates in unresectable patients are under 10% [1]. CCA is predominantly diagnosed in the 7th decade of life and affects 1-2 per 100,000 in the UK population [2], with a rising incidence worldwide [2–6].

The aetiology of CCA has not been clearly defined and is usually considered to be sporadic, although certain recognised predisposing factors have been identified, including primary sclerosing cholangitis (PSC; CCA occurs in up to 40% of PSC patients), gallstones, hepatitis C, cirrhosis, prolonged or recurrent biliary infection, liver fluke infection and carcinogen exposure. A number of mutations have been found in known oncogenes and tumour suppressor genes in CCA tissue specimens, however, the frequency of these mutations has been difficult to accurately assess and this information remains clinically unusable.

Indeterminate biliary strictures present a diagnostic challenge with multiple pathologies sharing similar clinical and radiological findings. In particular, the differentiation of CCA and PSC is difficult [7–9]. Endoscopic retrograde cholangiopancreatography (ERCP) following cross-sectional radiology for lesion assessment and biopsy has a sensitivity for malignancy of only 9–57%, whilst endoscopic ultrasound used in conjunction with fine needle aspiration for visualization and sampling has a sensitivity of ∼75% [10–13]. Novel cholangioscopic techniques have shown improved diagnostic accuracy compared to standard ERCP [14, 15]. Despite this, these invasive diagnostic procedures require highly-trained operators, are expensive to perform and can cause significant complications. Thus, non-invasive tests for differentiating CCA from benign pathologies are urgently needed.

Whilst efforts to discover effective blood-borne biomarkers for early detection are ongoing, the relative rarity of the disease and the frequent presence of cholestasis and cholangitis which can confound biomarker assays, have so far limited discovery efforts [16]. The best reported blood-borne tumour marker is carbohydrate antigen 19-9 (CA19-9), the sialylated Lewis (a) antigen, with the combination of CA19-9 and MRI/MRCP or ultrasound representing the most effective, cost-efficient and acceptable technique for screening and follow-up of CCA. However, ∼7% of the population who are Lewis (a) antigen negative do not produce CA19-9 and it is also often elevated in benign conditions presenting with similar indications, including PSC, primary biliary cirrhosis, cholestasis and cholangitis [17]. Indeed, serum bilirubin levels are an independent predictor of serum CA19-9 levels. The estimated sensitivity of CA19-9 in predicting CCA in the context of PSC is 38–89% with a specificity of 50–98% [18, 19]. Other reported serum markers include carcinoembryonic antigen (CEA) and carbohydrate antigen 125/mucin 16 (CA125/MUC16), although they are elevated in only ∼30% and 40–50% of cases, respectively [16].

One strategy to improve the diagnostic accuracy of CA19-9 may be to combine it with other biomarkers, as reported for bile galectin-3-binding protein (LGALS3BP) [20] and serum CEA [18]. Indeed, we have previously reported a combination of serum CA19-9, leucine-rich a2 glycoprotein 1 (LRG1) and interleukin 6 (IL6) that was capable of discriminating CCA from benign biliary strictures with an area under the receiver operating characteristics (ROC) curve (AUC) of 0.98, and independently of elevated bilirubin [21]. Other blood-based markers with potential to improve diagnostic accuracy include mucin 5AC (MUC5AC) [22–25], soluble fragment of cytokeratin 19 (CYFRA21.1) [26–28] and the pyruvate kinase isoenzyme M2 (PKM2) [29–31].

In a search for improved non-invasive diagnostic markers of CCA, we have investigated serum levels of CA19-9, LRG1, IL6, MUC5AC, CYFRA21.1 and PKM2 in a set of samples taken from patients diagnosed with CCA and PSC and tested combinations of these putative markers for differential diagnosis. Some of the candidates were also evaluated in pre-diagnosis samples from BTC and matched cancer-free controls to examine their value for early diagnosis.

## RESULTS

### Validation of a biomarker panel in patients with CCA compared with PSC

**Figure 1:**
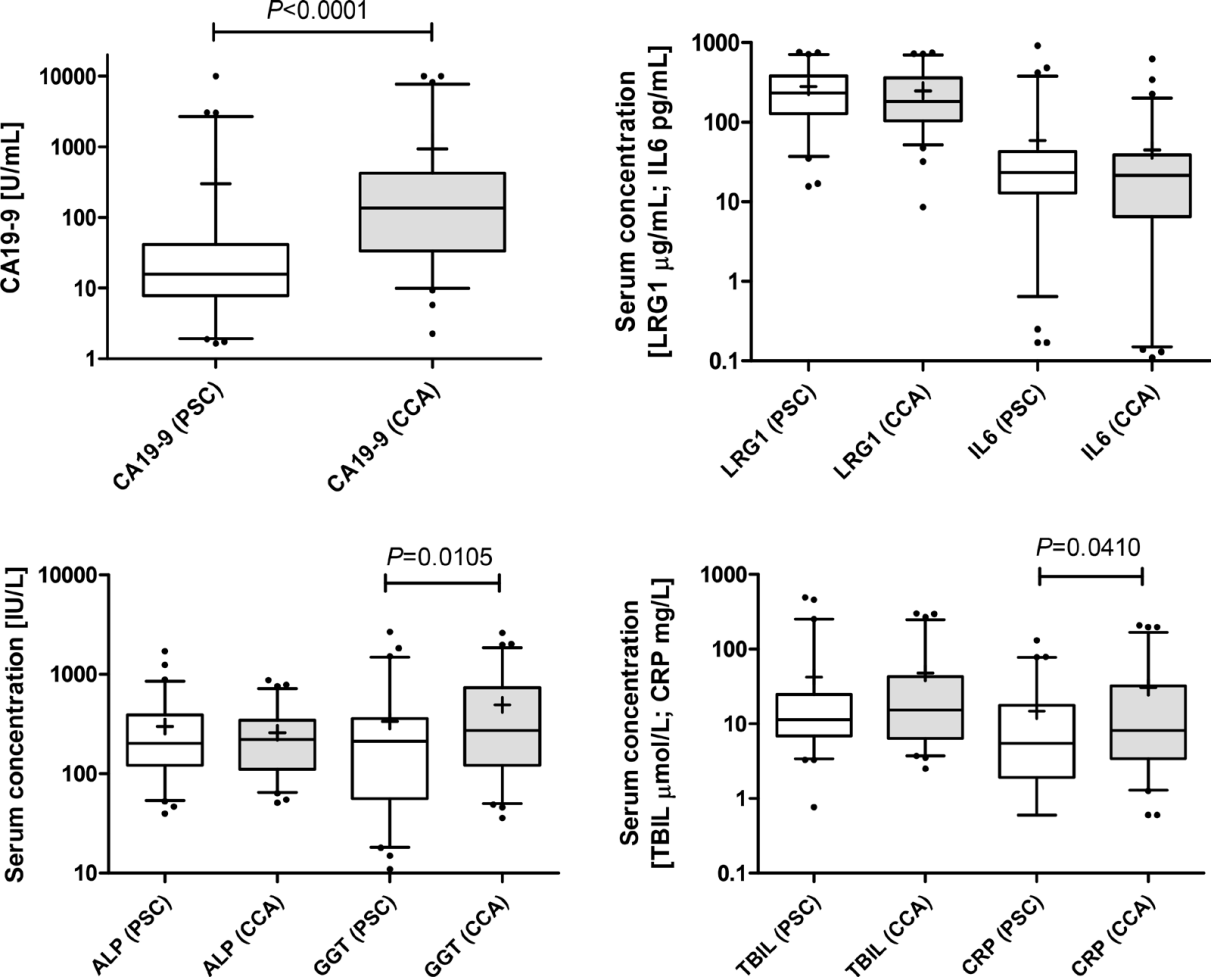
Box and whisker plots showing serum levels of CA19-9, LRG1 and IL6 in samples from PSC (*n* = 62; white boxes) and CCA patients (*n* = 66; grey boxes) ALP, GGT, TBIL and CRP were also measured in these samples. Whisker limits represent the 5th and 95th percentiles, the box limits represent the interquartile range, the horizontal line the median, and the ‘+’ the mean. *P* values (Mann-Whitney *U* test) are shown.

We previously reported a serum biomarker panel comprising of CA19-9, LRG1 and IL6 which was able to discriminate patients with CCA from benign biliary strictures with high accuracy [21]. Given that the sample size of the benign group used in this previous study was small (*n* = 13) and heterogeneous, testing of this biomarker panel in a larger, more homogeneous case control set was warranted. Thus, CA19-9, LRG1 and IL6 were measured in serum samples taken from 66 patients diagnosed with CCA and 62 diagnosed with PSC (Table 1). CA19-9 was confirmed as being significantly elevated in CCA cases (median 136.4 U/mL, interquartile range (IQR) 33.7–427.1 U/mL) compared to the PSC group (median 15.86 U/mL, IQR 7.9–41.5 U/mL; *P* < 0.0001). There was no significant difference in serum levels of LRG1 or IL6 between the groups (Figure 1). Markers of liver function and inflammation were also assessed. ALP and TBIL were unchanged between the CCA and PSC groups, whilst GGT and CRP were elevated in CCA *versus* PSC (*P* = 0.011 and *P* = 0.041, respectively) (Figure 1). ALP, TBIL, GGT and CRP levels were also positively correlated with one another (r > 0.34), and more so in the CCA group (r > 0.41). When cases were stratified into high and low CRP groups, using the median value as a cut-off, both CA19-9 and CRP maintained discriminatory ability, whilst GGT was only significant (*P* = 0.02) for the high CRP groups (data not shown).

**Table 1:** Demographics, clinical pathological data and biochemical profile of patient cohort

Variable		Cholangiocarcinoma (CCA)	Primary sclerosing cholangitis (PSC)	*P* value
Number of patients		66	62	
Male:Female (%)		37:29 (46%)	42:20 (33%)	0.205
Age (years)		65 (31–86)	52 (19–85)	< 0.0001
Stage (TNM)	I	3		
	II	13		
	IIIA	7		
	IIIB	15		
	IVA	13		
	IVB	6		
	Not specified	9		
Tumour classification =	Intrahepatic	12		
	Perihilar	38		
	Distal	4		
	Overlapping	6		
	Not specified	6		
CA19-9 (U/mL)		136.4 (2.3–10000)	15.9 (1.7–10000)	< 0.0001
CA19-9 > 37 U/mL (%)		49/66 (74%)	16/62 (26%)	< 0.0001
Total Bilirubin (μmol/L)		15.3 (2.5–300.2)	11.4 (0.8–493.2)	0.316
CRP (mg/L)		8.14 (0.6–208.0)	5.48 (0.6–131.6)	0.041
ALP (U/L)		222 (51–880)	203 (40–1714)	0.801
GGT (U/L)		273 (36–2623)	213 (11–2689)	0.011

= Tumour classification followed the guidelines of [39]. Median values with (range) are given, unless indicated otherwise. *P* values were determined using the Student *t*, Mann Whitney or Fishers exact test.

### Testing promising biomarkers for the differential diagnosis of CCA

**Figure 2:**
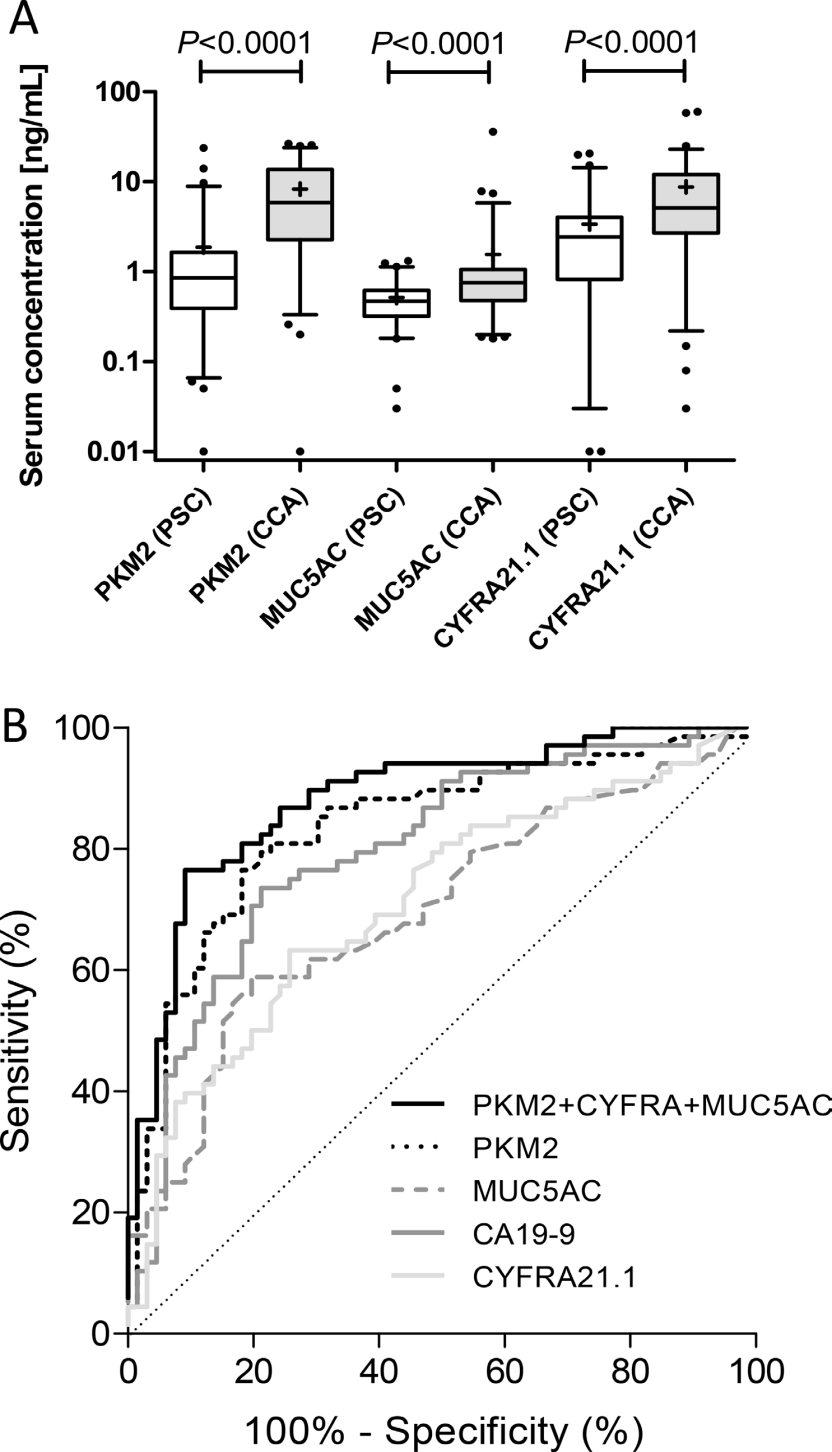
(**A**) Box and whisker plots of serum levels of PKM2, CYFRA21.1 and MUC5AC in samples from PSC (*n* = 62; white boxes) and CCA patients (*n* = 66; grey boxes). Whisker limits represent the 5th and 95th percentiles, the box limits represent the interquartile range, the horizontal line the median, and the ‘+’ the mean. *P* values (Mann-Whitney *U* test) are shown. (**B**) ROC curves of PKM2, CYFRA21.1, CA19-9 and MUC5AC, alone and in combination (see Table 3 for performance characteristics).

Potential biomarkers PKM2, MUC5AC and CYFRA21.1 previously assessed by our group and others [24, 27, 30, 32], were next tested in the study set. All three candidates were significantly elevated in malignant cases compared to benign controls (Figure 2A). PKM2 and MUC5AC maintained significance (*P* < 0.0001 and *P* < 0.001, respectively) irrespective of CRP level, whereas CYFRA21.1 was only significant (*P* < 0.0001) when comparing the high CRP groups (data not shown). PKM2 gave 75.8% sensitivity and 82.3% specificity at a cut-off of > 2.2 ng/mL, MUC5AC gave 60.6% sensitivity and 82.3% specificity at a cut-off of > 0.67 ng/mL and CYFRA21.1 gave 65.2% sensitivity and 75.8% specificity at a cut-off of > 4.0 ng/mL (Table 3; Figure 2B). When intrahepatic cases (*n* = 12) were compared against extrahepatic CCA cases (*n* = 46), only PKM2 was significantly different between the groups (*P* = 0.022), being elevated in intrahepatic cases stage (Supplementary Figure 1). This suggests that any difference in treatment between the two groups, e.g. endoscopic evaluation and stenting, was not a major confounding factor, particularly involving an inflammatory response. Notably, none of the tested candidates showed significant differences in serum levels dependent upon TNM stage (Supplementary Figure 2).

**Table 2:** Clinical and sample characteristics of UKCTOCS BTC and control study set

		Biliary tract cancer	Cancer-free controls	*P*-value
Number of cases		55	91	
Number of samples		89	91	
Median age and range (years)		65 (52–75)	62 (50–77)	0.0004
Median time to spin and range (hours)		22.8 (0.5–46.0)	22.6 (1.5–46.0)	0.965
Median time from sample collection to diagnosis and range (months)		31.5 (0.9–66.6)		
Tumour site:	Intrahepatic	29		
	Extrahepatic	4		
	Gall bladder	12		
	Overlapping	10		
Pre-diagnosis time group:	0–1 y	10		
	1–2 y	26		
	2–3 y	19		
	> 3 y	34		

**Table 3:** Performance of single markers for discriminating CCA and PSC

Biomarker(s)	Cut-off	Sensitivity % (95% CI)	Specificity % (95% CI)	AUC (95% CI)
CA19-9 (U/mL)	> 37.0	74.2 (62.0–84.2)	74.2 (61.5–84.5)	0.789 (0.71–0.87)
LRG1 (μg/mL)	> 57.5^*^	4.5 (0.9–12.1)	91.9 (82.2–97.3)	0.562 (0.461–0.662)
IL6 (pg/mL)	> 48.4^*^	80.3 (68.7–89.1)	17.7 (9.2–29.5)	0.543 (0.443–0.643)
PKM2 (ng/mL)	> 2.2	75.8 (63.6–85.5)	82.3 (70.5–90.8)	0.839 (0.768–0.91)
CYFRA21.1 (ng/mL)	> 4.0	65.2 (52.4–76.5)	75.8 (63.3–85.8)	0.732 (0.645–0.819)
MUC5AC (ng/mL)	> 0.67	60.6 (47.8–72.4)	82.3 (70.5–90.8)	0.72 (0.631–0.809)

CI = confidence interval.^*^Cut-offs used in previous study [21].

### Combined logistic regression models

Combinations of candidates were next tested, using logistic regression to generate models. The AUC for a model combining CA19-9, LRG1 and IL6 was only 0.63 (Table 4), and so failed to validate our previous findings [21]. Combining PKM2 with CYFRA21.1, MUC5AC or CA19-9 gave AUCs of 0.85–0.88 with sensitivities ranging from 64-71% at 90% specificity, and PKM2 was the marker that featured most commonly in the top models. Combining CYFRA21.1 and MUC5AC, did not significantly improve on using either candidate marker alone (Table 4). The best-performing 3-variable models ([PKM2, MUC5AC, CYFRA21.1] and [PKM2, CYFRA21.1, TBIL]) had AUCs of 0.90 (Figure 2B) and 0.87 with sensitivities of 75.8% and 80.3%, respectively, at 90% specificity (Table 4). Addition of CA19-9 to the former model did not improve the AUC, and indeed, CA19-9 did not feature prominently in the best models. The best-performing model combined PKM2, MUC5AC, CYFRA21.1 and GGT with a sensitivity of 81.8%, specificity of 90% and an AUC of 0.90.

**Table 4:** Performance of selected single and multivariate logistic regression models for discriminating CCA and PSC ranked in order of sensitivity at 90% specificity

Model	Sensitivity (%)	Specificity (%)	AUC
PKM2, MUC5AC, CYFRA21.1, GGT	81.8	90.0	0.903
PKM2, CYFRA21.1, TBIL	80.3	90.0	0.868
PKM2, MUC5AC, CYFRA21.1, CRP	77.3	90.0	0.907
PKM2, MUC5AC, CYFRA21.1, ALP	77.3	90.0	0.899
PKM2, MUC5AC, CYFRA21.1, TBIL	75.8	90.0	0.899
LRG1, PKM2, MUC5AC, CYFRA21.1	75.8	90.0	0.899
PKM2, MUC5AC, CYFRA21.1	75.8	90.0	0.899
CA19.9, PKM2, MUC5AC, CYFRA21.1	75.8	90.0	0.897
PKM2, MUC5AC, CRP, ALP	74.2	90.0	0.895
CA19.9, PKM2, TBIL	74.2	90.0	0.867
CA19.9, PKM2, CYFRA21.1	72.7	90.0	0.87
PKM2, CYFRA21.1	71.2	90.0	0.869
CA19.9, PKM2, MUC5AC, CRP	69.7	90.0	0.891
LRG1, PKM2, MUC5AC, GGT	69.7	90.0	0.888
PKM2, CYFRA21.1, ALP	69.7	90.0	0.87
PKM2, CRP, GGT	69.7	90.0	0.866
PKM2, GGT	68.2	90.0	0.866
PKM2, CRP	68.2	90.0	0.85
PKM2, TBIL	68.2	90.0	0.848
PKM2, MUC5AC, CRP	66.7	90.0	0.889
CA19.9, PKM2	66.7	90.0	0.854
CA19.9, PKM2, MUC5AC	65.2	90.0	0.882
PKM2, MUC5AC	63.6	90.0	0.88
PKM2	60.6	90.0	0.842
CA19.9	50.0	90.0	0.801
CA19.9, CYFRA21.1	47.0	90.0	0.765
MUC5AC, CYFRA21.1	45.5	90.0	0.78
CYFRA21.1	40.9	90.0	0.73
CA19.9, LRG1, IL6	36.4	90.0	0.637
MUC5AC	30.3	90.0	0.728

To address possible confounding effects of biliary obstruction on biomarker performance, samples were stratified into low and high TBIL groups based on the median as a cut-off (12.7 μmol/L) and diagnostic performance assessed for the two groups. For the low TBIL group (CCA *n* = 31; PSC *n* = 32), the performance of individual markers and combined models were generally better compared to the full study set, with the best model (PKM2; CYFRA21.1; MUC5AC) providing 87.1% sensitivity at 90% specificity, with PKM2 again featuring prominently in the top models (Supplementary Table 1). Model performances for the high TBIL group (CCA *n* = 35; PSC *n* = 30) were generally lower compared to the full set, particularly those incorporating CYFRA21.1 (Supplementary Table 1). Thus the sensitivity of the PKM2; CYFRA21.1; MUC5AC model decreased to 65.7% at 90% specificity for the high TBIL group. The top models featured PKM2 and CRP or GGT and gave the same sensitivity of 74.3%. Conversely, models using CA19-9 or CRP showed modest improvement in performance in the high *versus* low TBIL groups.

### Regression tree and random forest analysis

Given that the discriminatory power of some of the markers changed when samples were stratified by TBIL or CRP, we wanted to test algorithms more suited to model any interactions between variables. A Classification and Regression Tree model gave 95% sensitivity at 71% specificity with an AUC of 0.83 using PKM2, CYFRA21.1 and CA19-9 in the model (Supplementary Figure 3). Logistic regression models were also compared with the random forest ensemble learning method for classification. Variable selection within the random forest algorithm reported proteins PKM2, CA19.9, CYFRA21.1, GGT and CRP as those having highest variable importance, whilst selection within the logistic regression reported PKM2, MUC5AC, CYFRA21.1, CRP, ALP and GGT. The performance of the two algorithms was similar: AUC = 0.912 (sensitivity of 75.8% at specificity of 90%) for the random forest *versus* AUC = 0.909 (sensitivity of 81.8% at specificity of 90%) for the logistic regression. The results indicate that logistic regression remains a trustworthy classification algorithm for CCA *versus* PSC using the biomarkers reported herein.

### Diagnostic performance of biomarkers in pre-diagnosis samples

To assess the potential of the markers for early diagnosis of BTC, assays for TBIL, ALP, GGT, CRP, CA19-9, LRG1, IL6 and PKM2 were conducted on a set of 89 pre-diagnosis serum samples taken from 55 cases of BTC and 91 matched cancer-free controls identified from the UKCTOCS biobank (Table 2). The median time from sample collection to diagnosis was 31.5 months. When all samples were considered, CA19-9 (*P* = 0.002), ALP (*P* = 0.006), GGT (*P* = 0.039) and CRP (*P* = 0.0007) were significantly elevated in BTC pre-diagnosis samples compared to cancer-free controls, as was the proportion of samples with CA19-9 levels > 37 U/mL (*P* = 0.038) (Supplementary Table 2). TBIL, LRG1, IL6 and PKM2 were not significantly elevated. CA19-9 provided 17% sensitivity at a specificity of 93% using the standard cut-off value of > 37 U/mL with an optimised cut-off giving 53% sensitivity and 69% specificity (Table 5).

**Table 5:** Univariate and multivariate biomarker performance in 0-1 year time group and for all pre-diagnosis samples

Time group (years)	Biomarker(s)	Sensitivity (95% CI)	Specificity (95% CI)	AUC (95% CI)
0–1 (*n* ***=*** 10)	CA19-9	80.0 (44.4–97.5)	69.2 (58.7–78.5)	0.770 (0.577–0.963)
	TBIL	70.0 (34.8–93.3)	69.2 (58.7–78.5)	0.651 (0.458–0.845)
	PKM2	60.0 (26.2–87.8)	50.0 (39.9–61.2)	0.525 (0.358–0.693)
	CRP	70.0 (34.8–93.3)	73.6 (63.3–82.3)	0.728 (0.525–0.930)
	GGT	90.0 (55.5–99.8)	65.9 (55.3–75.6)	0.793 (0.628–0.958)
	ALP	80.0 (44.4–97.5)	63.7 (53.0–73.6)	0.771 (0.636–0.906)
	CA19-9; PKM2	50.0 (20.0–80.0)	90.1 (74.7–100)	0.781 (0.580–0.983)
	CA19-9; CRP	60.0 (30.0–90.0)	90.1 (58.2–100)	0.812 (0.615–1.00)
	CA19-9; ALP	60.0 (30.0–90.0)	90.1 (40.7–100)	0.824 (0.661–0.988)
	CA19-9; PKM2; LRG; IL6	60.0 (20.0–80.0)	90.1 (35.2–100)	0.803 (0.627–0.979)
All (*n* ***=*** 89)	CA19-9	52.8 (41.9–63.5)	69.2 (58.7–78.5)	0.633 (0.552–0.715)
	TBIL	42.7 (32.3–53.6)	67.0 (56.4–76.5)	0.505 (0.419–0.590)
	PKM2	59.6 (48.6–69.8)	50.6 (39.9–61.2)	0.529 (0.444–0.614)
	CRP	60.7 (49.8–70.9)	64.8 (54.1–74.6)	0.646 (0.565–0.727)
	GGT	49.4 (38.7–60.3)	71.4 (61.0–80.4)	0.589 (0.505–0.673)
	CA19-9; CRP	33.7 (14.6–48.3)	90.1 (78.0–95.6)	0.689 (0.611–0.767)
	CA19-9; PKM2; LRG1; IL6	29.2 (11.2–40.5)	90.1 (75.8–95.6)	0.640 (0.559–0.722)

**Figure 3:**
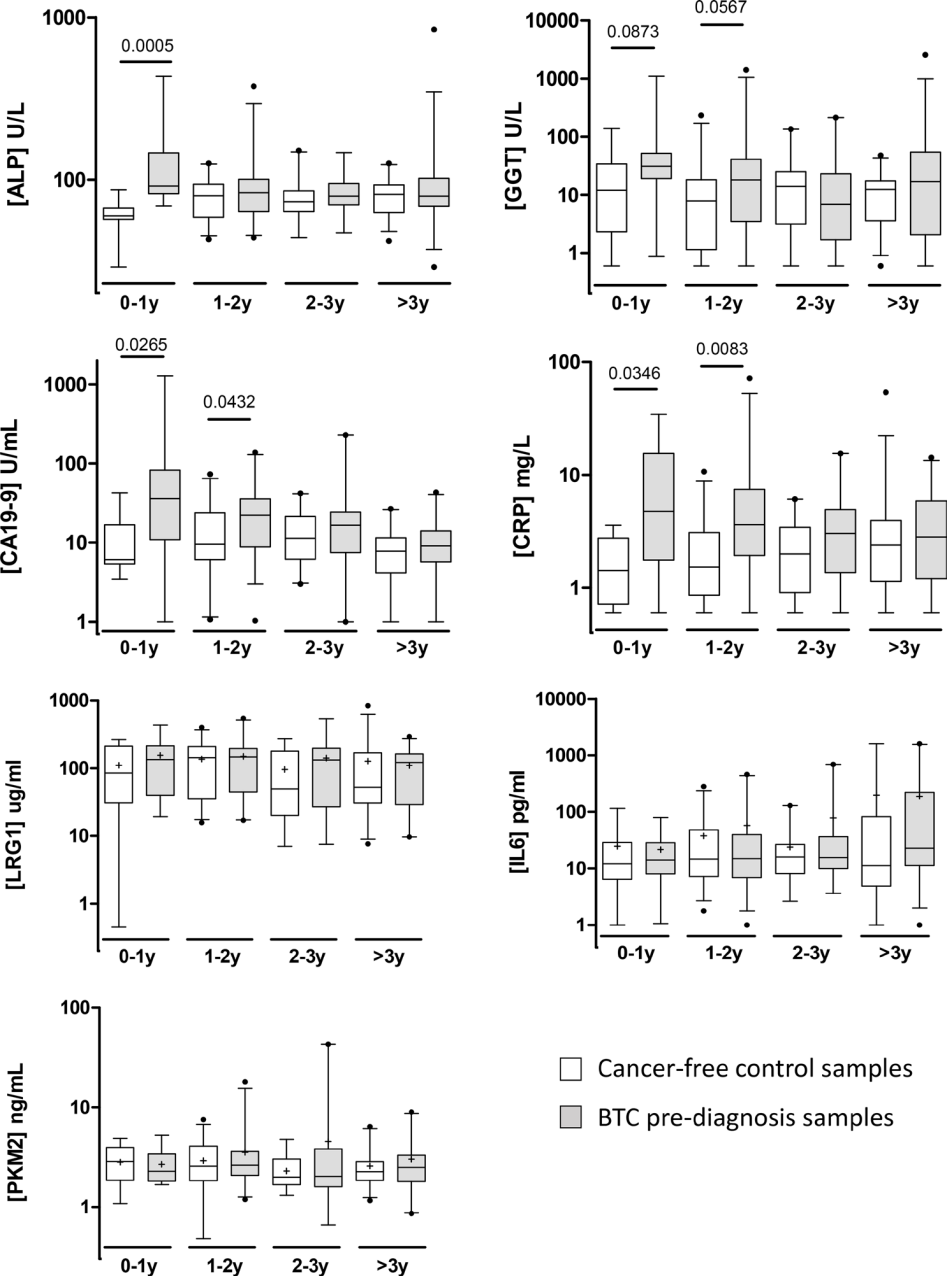
Box and whisker plots showing serum levels of ALP, GGT, CA19-9, CRP, PKM2, LRG1 and IL6 for cancer-free controls (*n* = 91; white boxes) and BTC cases (*n* = 89; grey boxes) in UKCTOCS pre-diagnosis samples Samples are grouped into different time-to-diagnosis groups. Whisker limits represent the 5th and 95th percentiles, the box limits represent the interquartile range, the horizontal line the median, and the ‘+’ the mean. Case and control groups were compared using the Mann-Whitney *U* test; *P* values are shown above the plots.

In stratifying the samples by time to diagnosis, TBIL, LRG1, IL6 and PKM2 remained unchanged across the time groups. ALP was elevated only within 1 year of diagnosis, whilst CA19-9, CRP and GGT displayed increasing levels towards diagnosis, although the differences between cases and controls for GGT narrowly failed significance (Figure 3). CRP and CA19-9 were also significantly elevated 1-2 years before diagnosis, but not beyond. Combining LRG1, IL6 and PKM2 with CA19-9 did not significantly improve the AUC compared to CA19-9 alone, either for all samples or just those taken within 1 year of diagnosis (Table 4). Combining CRP and ALP with CA19-9 also did not significantly improve the AUC compared to CA19-9 alone, either for all samples or just those within 1 year to diagnosis.

## DISCUSSION

There are relatively few studies that have tested circulating biomarkers for CCA. This study aimed to validate previously proposed biomarkers and to investigate their use for early detection in the pre-diagnosis setting. Our previously reported biomarker panel of CA19-9, IL6 and LRG1 [21] did not perform as well in this larger, more homogeneous cohort of CCA and PSC patients. The inflammatory cytokine IL6 was previously found to be increased in the circulation of CCA patients [33–35] and was reported to synergistically induce the expression of LRG1 along with other inflammatory cytokines [36]. In the present cohort, IL6 and LRG1 were positively correlated (r = 0.339 in the CCA group), so it is perhaps unsurprising that we found no complementarity in using IL6 and LRG1 together. It is important to note that our previous study used a relatively small sample size, particularly of benign cases, which were heterogeneous in nature and displayed inconsistency in the levels of inflammatory markers between the groups. This is likely to have contributed to an exaggerated performance of IL6 and LRG1 in our previous work.

PKM2, MUC5AC and CYFRA21.1 however, were all found to distinguish CCA from the PSC group. Pyruvate kinase is an enzyme regulating the final rate-limiting step of glycolysis, with the PKM2 isoform overexpressed in many cancer types and proposed to promote aerobic glycolysis (the Warburg effect). We previously showed that bile and plasma PKM2 were elevated in BTC and was a predictor of tumour progression [30]. The results presented herein corroborate PKM2 as a biomarker for the differential diagnosis of CCA and PSC. When used in combination with MUC5AC and CYFRA21.1, PKM2 added to diagnostic performance; at 90% specificity, the sensitivity increased from 60.6% for PKM2 alone to 75.8% for the 3-marker panel and was further improved to 81.8% by addition of GGT. MUC5AC is a member of the membrane-bound and secreted epithelial mucin glycoprotein family and a previously reported serum marker of CCA [22, 25, 37]. In support of this, we showed that MUC5AC serum levels were increased in CCA *versus* PSC and contributed to the diagnostic performance of the biomarker panel. Serum levels of the cytokeratin 19 fragment CYFRA21.1 also had diagnostic potential, again supporting previous reports [27, 28, 38]. Since both CYFRA21.1 and MUC5AC are involved in remodelling of the extracellular milieu, their elevation in CCA may suggest epithelial-mesenchymal transition and increased metastatic potential. However, neither CYFRA21.1, MUC5AC or any of the other candidates tested were significantly altered depending on TNM stage.

Notably, CA19-9 was not in any of the best biomarker panels and added little to diagnostic performance when used in combination. This may be indicative of an association with inflammation, and indeed the inflammatory marker CRP was also elevated in the CCA group. However, CA19-9, PKM2 and MUC5AC could similarly classify CCA from the benign group independently of CRP, whilst CYFRA21.1 was discriminatory only for the high CRP cases. Thus, the markers appear to be specific, performing well irrespective of underlying inflammatory status. Biliary obstruction is also known to affect tumour biomarker measurements in the context of CCA. We showed that whilst CA19-9, PKM2, CYFRA21.1 and MUC5AC could discriminate CCA cases independently of total bilirubin, the sensitivities of the biomarker panels at fixed specificity were reduced in high bilirubin cases, particularly those panels including CYFRA21.1. This suggests TBIL as a confounding factor for the differential diagnosis of CCA and should be taken into consideration for future validation studies. The exact causes of the interference are as yet unclear.

We next investigated a subset of the markers in the pre-diagnosis setting to assess their worth for early detection of BTC (CCA and gall bladder cancer). Rising serum levels of CA19-9, CRP and ALP were apparent in BTC cases towards diagnosis, and all 3 markers were significantly (*P* < 0.05) different between BTC and non-cancer controls up to two years before diagnosis. For CRP and ALP, this suggests an inflammatory response is present and that liver/gall bladder function are affected well in advance of diagnosis. However, these changes are not likely to be specific, and indeed for ALP, an elevation was not apparent in diagnosed CCA cases *versus* PSC. For CA19-9, sensitivity was 50% at 90% specificity (0–1 year time group), and was not significantly improved by adding any other marker. Thus, it would be unsuitable for screening the general population, although may have some utility for screening high-risk groups. Disappointingly, LRG1, IL6 and PKM2 failed to show discriminatory potential. This is somewhat contrary to the elevation of LRG1 and IL6 in association with inflammation, since CRP was elevated in these prediagnosis cases. Whilst this suggests some specificity in the inflammatory response to BTC, we cannot rule out the possibility that CRP was elevated by chance in the BTC group due to other unreported conditions. The unaltered levels of PKM2 prior to diagnosis may suggest that PKM2 elevation in the circulation occurs only after the tumour is established and in response to the increased metabolic demand of rapidly dividing cells. There are several weaknesses to our evaluation of markers of BTC in this pre-diagnosis set; the number of cases was relatively small, particularly in the 0-1 year time group, details of tumour stage at diagnosis were not available, only post-menopausal women were sampled and appropriate benign controls were not available.

In conclusion, a panel of previously identified circulating biomarkers (CA19-9, LRG1 and IL6) aimed at the differential diagnosis of CCA failed to validate in this refined cohort of patients. Other promising markers (PKM2, CYFRA21.1 and MUC5AC) gave respectable classification performances in discriminating cancer cases from relevant benign controls and were independent of CRP and tumour site. The combination of PKM2, CYFRA21.1, MUC5AC and GGT effectively discriminated CCA from PSC with a sensitivity of 81.8% and specificity at 90%, and warrants validation in a prospective trial. A *post hoc* power calculation using PKM2 alone showed that 55 samples per group would be sufficient to provide a power of 0.95 at an alpha of 0.05 and combination models using decision tree or random forest analysis performed similarly to logistic regression models. Thus, our models appears to be robust and would be reproducible in a larger cohort. In pre-diagnosis samples of BTC, CA19-9 was significantly elevated up to 2 years before diagnosis, but was not useful alone or in combination with other markers for accurate early detection. CYFRA21.1 and MUC5AC are yet to be tested in the pre-diagnosis setting.

## MATERIALS AND METHODS

### Patient samples

The study was conducted following ethical approval by the Joint UCL/UCLH Research Ethics Committee A (Ref. 06/Q0152/106) and Hannover Medical School Ethics Committee. Written informed consent was obtained from all patients. Blood samples were collected by venepuncture into Vacutainer SST tubes (BD, Franklin Lakes, NJ, USA) from patients with a confirmed diagnosis of CCA (*n* = 66) or PSC (*n* = 62) at University College London Hospital, the Royal Free Hospital London and the Hannover Medical School, between 01/2009 and 06/2015. Tumour site and classification followed the guidelines of [39]. Bloods were taken from CCA and PSC patients with either no prior endoscopic intervention, weeks to months after endoscopic treatment with ERCP (dilatation or stent insertion) or just before ERCP. All had a current stricture when included into the study. To 06/2015, none of the PSC patients were diagnosed with CCA. Blood was allowed to clot for 1 hr and separated by centrifugation at 2,200 rpm for 10 min at 4°C. Serum was recovered, aliquoted and stored at –80°C until further use. Baseline patient demographics and clinical pathological data for this case control set are shown in Table 1.

Serum samples predating diagnosis of BTC and matched controls came from post-menopausal women recruited to the UK Collaborative Trial of Ovarian Cancer Screening (UKCTOCS) and were collected according to a standard operating procedure [40, 41]. This nested case-control study within UKCTOCS was approved by the Joint UCL/UCLH Research Ethics Committee A (Ref. 05/Q0505/57). Informed consent was obtained from all volunteers and data was anonymised. Using volunteer NHS numbers, the Health and Social Care Information Centre cancer and death registers were interrogated for UKCTOCS participants who were subsequently diagnosed with CCA or gall bladder cancer (ICD10 codes C22.1/9, C23, C24.0/8). There were 55 cases of BTC identified (prior to Feb 2009) with 89 samples taken from these cases which were categorised into pre-diagnosis time groups (Table 2). Cases were matched with cancer-free controls (*n* = 91) by age (± 5 years), regional collection centre (same) and collection date (same).

### Serum assays

Standard blood tests including liver biochemistry (total bilirubin (TBIL), alkaline phosphatase (ALP), gamma-glutamyl transferase (GGT)), C-reactive protein (CRP) and CA19-9 (Cobas CA19-9 CLIA; Roche and Fujirebio Diagnostics) were carried out at the Clinical Biochemistry service of UCLH. Samples were evaluated for the other candidate biomarkers using the following commercial ELISA kits at the dilutions specified and with the indicated intra-assay CVs: human LRG1 ELISA kit (IBL International, Hamburg, Germany; 1:2000; CV = 5.5%), human IL-6 ELISA Kit (Pierce Biotechnology, Rockford, IL, USA; 1:5; CV = 6.7%), human PKM2 ELISA kit (Cloud-Clone Corp. Wuhan, China; 1:10; CV = 10.3%), human cytokeratin fragment antigen 21-1 (CYFRA21.1) ELISA Kit (Cusabio, Wuhan, China; 1:5; CV = 8.1%) and human MUC5AC ELISA Kit (Elabscience, Bethesda, MD, USA; 1:2; CV = 17.4%).

### Statistical analysis and biomarker modelling

Statistical analyses were carried out using GraphPad Prism V5 and R 3.4.1 software packages. Continuous data between clinical groups were compared using the Mann-Whitney *U*-test for non-parametric data and the Student *t*-test for normally distributed data. The recommended clinical cut-off of 37 U/mL was used for CA19-9 and defined cut-offs for LRG1 (57.5 μg/mL) and IL6 (48.4 pg/mL) were the optimal points for sensitivity and specificity from a previous study [21]. Multivariable logistic regression analysis was used to examine the inter-relationship between serum biomarkers, liver function tests, biliary obstruction and cancer likelihood. The number of variables in models was restricted to ensure performance of models was reproducible without risk of overfitting; making sure that the one in ten rule was satisfied [42, 43]. ROC curves were generated and AUCs obtained and compared for single and combined biomarker models for discriminating CCA from PSC or between BTC cases and cancer-free controls in different time to diagnosis time groups for the UKCTOCS cohort. DeLong’s test was used to assess differences between ROC curves. *P* values < 0.05 were considered significant. Sensitivities at fixed specificity (90%) were calculated for all biomarkers and combinations. Classification and Regression Tree analysis was performed using the RPART package in R. Logistic regression models were also compared with the random forest ensemble learning method for classification [44] using R packages: ‘randomForest’ for implementation of the random forest algorithm, ‘VSURF’ for variable selection within the random forest and ‘MASS’ for the variable selection using the Akaike Information Criterion for the logistic regression models.

## SUPPLEMENTARY MATERIALS FIGURES AND TABLES



## Abbreviations

CA19-9: carbohydrate antigen 19-9
LRG1: leucine-rich a2-glycoprotein
IL6: interleukin 6
PKM2: pyruvate kinase M2
CYFRA21.1: cytokeratin 19 fragment
MUC5AC: mucin 5AC
BTC: biliary tract cancer
PSC: primary sclerosing cholangitis
AIC: autoimmune cholangiopathy
UKCTOCS: United Kingdom Collaborative Trial of Ovarian Cancer Screening
CCA: cholangiocarcinoma
ERCP: endoscopic retrograde cholangiopancreatography
TBIL: total bilirubin
ALP: alkaline phosphatase
GGT: gamma-glutamyl transferase
CRP: C-reactive protein
ROC: receiver operating characteristic
AUC: area under the ROC curve
