# Supplementary material for: Identification of a serum biomarker panel for the differential diagnosis of cholangiocarcinoma and primary sclerosing cholangitis
Source: Oncotarget. 2018 Apr 3;9(25):17430–42. doi: 10.18632/oncotarget.24732 (PMC5915126; doi:10.18632/oncotarget.24732)
Supplement: Supplementary file 1 [file oncotarget-09-17430-s001.pdf]

Identification of a serum biomarker panel for the differential diagnosis of cholangiocarcinoma and primary sclerosing cholangitis

SUPPLEMENTARY MATERIALS

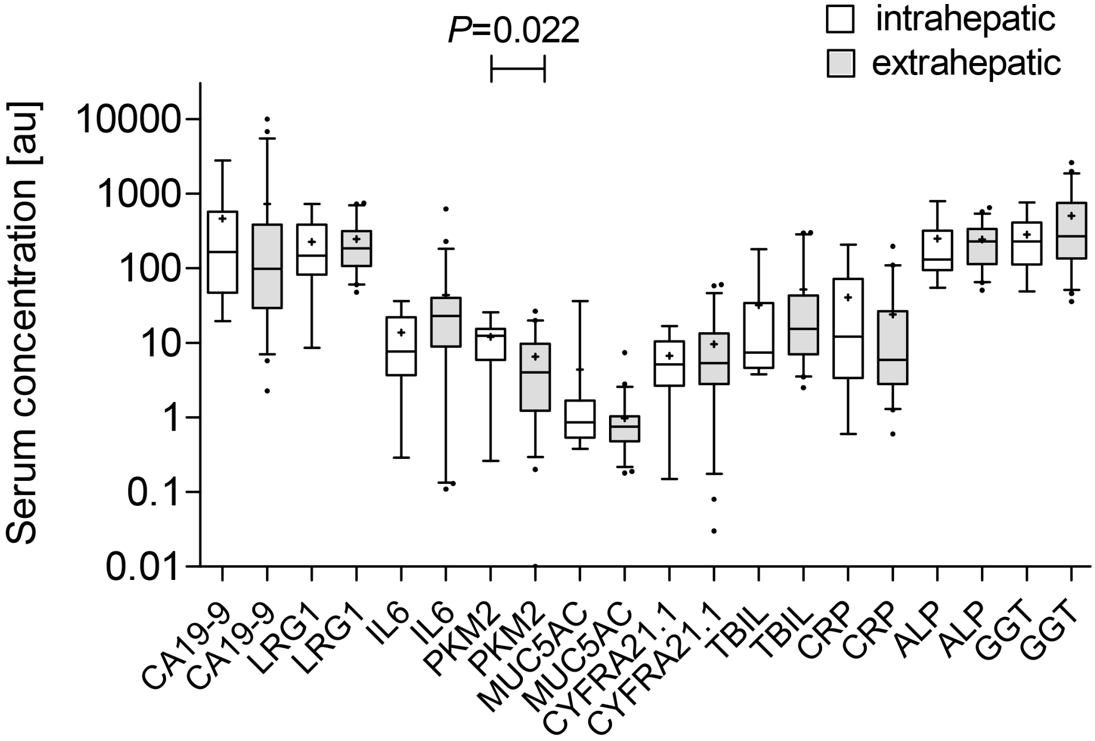

Supplementary Figure 1: Serum biomarker levels in CCA cases by tumour location: intrahepatic (*n* = 12) versus extrahepatic (perihilar, distal and overlapping; *n* = 46).

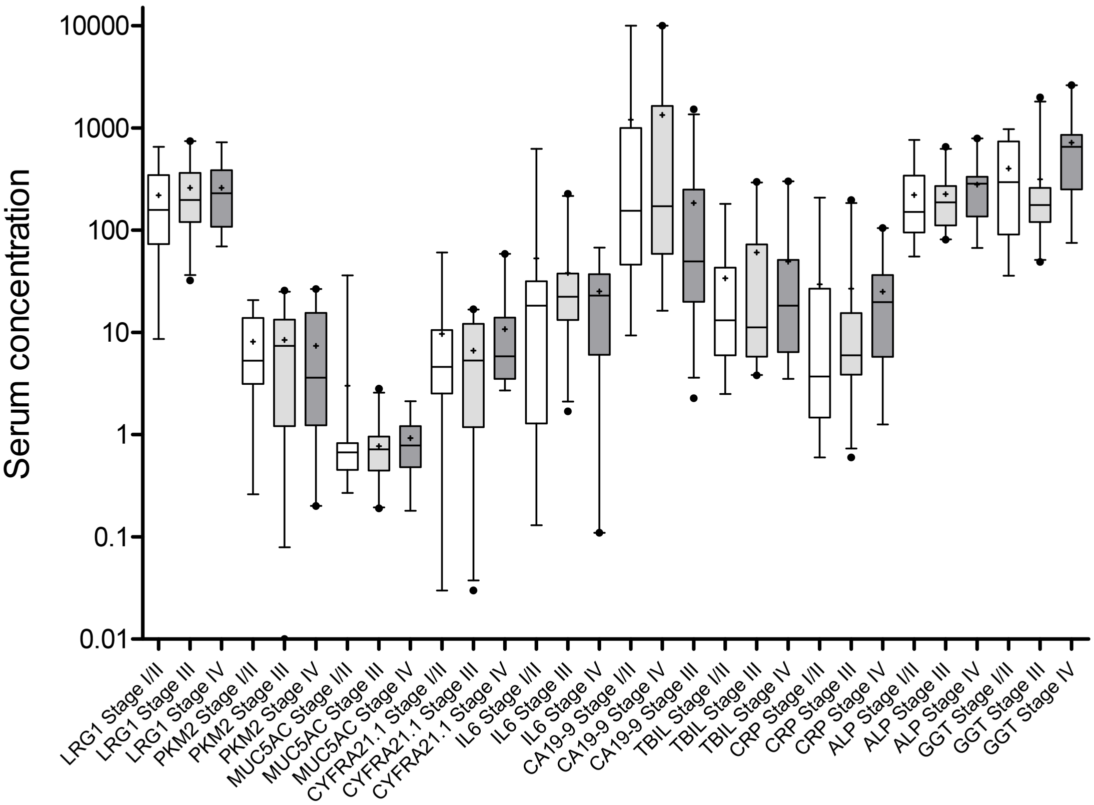

Supplementary Figure 2: Serum biomarker levels in CCA cases by TNM stage (stage I/II (*n* = 16), stage III (*N* = 22) and stage IV (*n* = 19)).

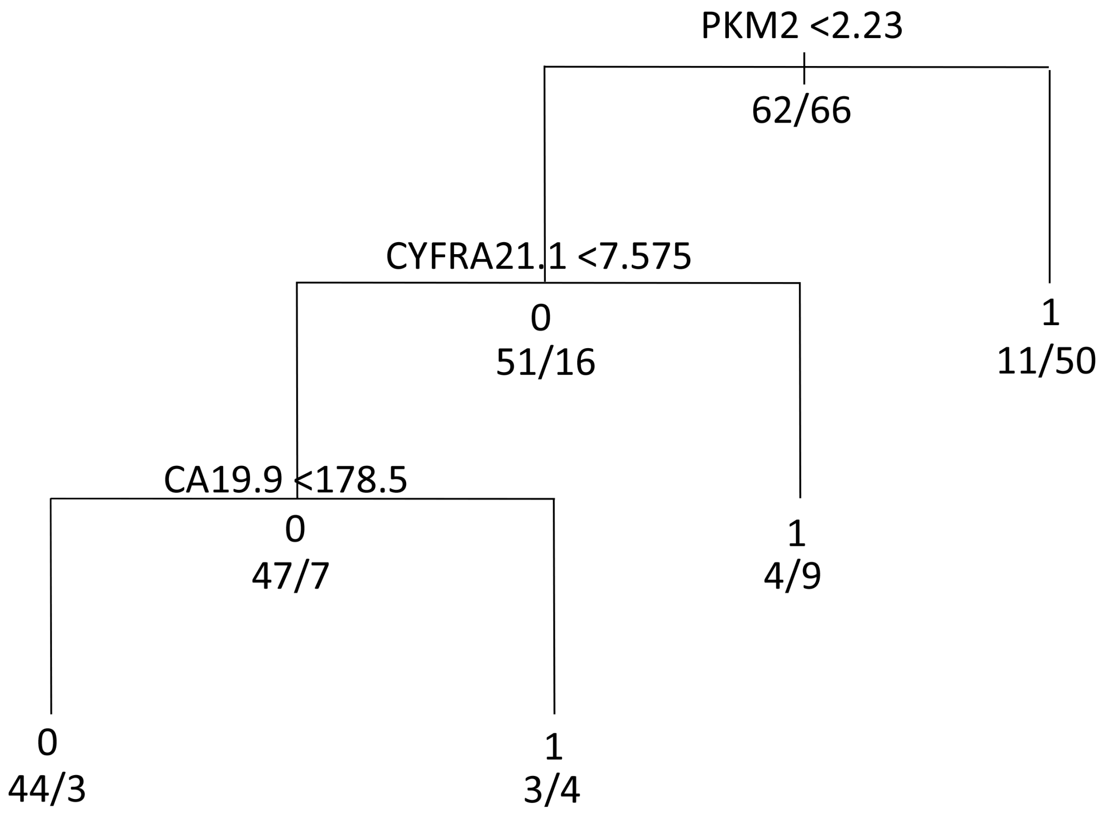

Supplementary Figure 3: Classification tree for discrimination of CCA and PSC.

Supplementary Table 1: Biomarker model performance in CCA and PSC cases stratified by low TBIL (< 12.7 µmol/L; CCA *n* = 31; PSC *n* = 32) and high TBIL (> 12.7 µmol/L; CCA *n* = 35; PSC *n* = 30)

	TBIL low (< 12.7 µmol/L)			TBIL high (> 12.7 µmol/L)			
**Model**	**Sensitivity**	**Specificity**	**AUC**	**Sensitivity**	**Specificity**	**AUC**	**Difference in sensitivity**
PKM2, MUC5AC, CYFRA21.1	0.871	0.9	0.918	0.657	0.9	0.89	–0.214
PKM2, CYFRA21.1, GGT	0.871	0.9	0.902	0.743	0.9	0.875	–0.128
PKM2, CYFRA21.1, CRP	0.871	0.9	0.901	0.743	0.9	0.884	–0.128
LRG1, PKM2, CYFRA21.1	0.871	0.9	0.899	0.6	0.9	0.845	–0.271
PKM2, CYFRA21.1	0.871	0.9	0.899	0.6	0.9	0.842	–0.271
PKM2, CYFRA21.1, CA19.9	0.839	0.9	0.902	0.571	0.9	0.839	–0.268
PKM2, TBIL	0.806	0.9	0.883	0.6	0.9	0.829	–0.206
PKM2, TBIL, CRP	0.806	0.9	0.883	0.714	0.9	0.869	–0.092
PKM2, CA19.9, TBIL	0.806	0.9	0.878	0.571	0.9	0.827	–0.235
PKM2, CA19.9, CRP	0.774	0.9	0.887	0.686	0.9	0.854	–0.088
PKM2, CA19.9, GGT	0.774	0.9	0.885	0.714	0.9	0.874	–0.06
PKM2, CA19.9	0.774	0.9	0.879	0.571	0.9	0.823	–0.203
PKM2, ALP, GGT	0.774	0.9	0.876	0.714	0.9	0.871	–0.06
PKM2, CRP	0.774	0.9	0.873	0.714	0.9	0.865	–0.06
PKM2	0.774	0.9	0.872	0.571	0.9	0.823	–0.203
PKM2, CRP, ALP	0.774	0.9	0.869	0.743	0.9	0.871	–0.031
PKM2, MUC5AC, IL6	0.742	0.9	0.907	0.657	0.9	0.867	–0.085
PKM2, MUC5AC, ALP	0.71	0.9	0.925	0.629	0.9	0.861	–0.081
PKM2, MUC5AC, CRP	0.71	0.9	0.904	0.743	0.9	0.901	0.033
PKM2, CRP, GGT	0.71	0.9	0.88	0.686	0.9	0.888	–0.024
PKM2, GGT	0.71	0.9	0.88	0.743	0.9	0.881	0.033
LRG1, PKM2, GGT	0.71	0.9	0.875	0.714	0.9	0.883	0.004
PKM2, TBIL, GGT	0.71	0.9	0.875	0.743	0.9	0.879	0.033
MUC5AC, CYFRA21.1, CRP	0.71	0.9	0.814	0.371	0.9	0.808	–0.339
MUC5AC, CYFRA21.1	0.71	0.9	0.785	0.286	0.9	0.776	–0.424
PKM2, MUC5AC, CA19.9	0.677	0.9	0.913	0.6	0.9	0.858	–0.077
PKM2, MUC5AC	0.645	0.9	0.91	0.657	0.9	0.862	0.012
PKM2, MUC5AC, GGT	0.645	0.9	0.91	0.714	0.9	0.887	0.069
LRG1, PKM2, MUC5AC	0.613	0.9	0.913	0.657	0.9	0.861	0.044
PKM2, MUC5AC, TBIL	0.613	0.9	0.907	0.657	0.9	0.862	0.044
CYFRA21.1, GGT	0.613	0.9	0.777	0.171	0.9	0.689	–0.442
CYFRA21.1, CRP	0.581	0.9	0.793	0.343	0.9	0.702	–0.238
CYFRA21.1, CA19.9	0.581	0.9	0.782	0.143	0.9	0.748	–0.438
CYFRA21.1	0.581	0.9	0.78	0.257	0.9	0.691	–0.324
LRG1, CYFRA21.1	0.581	0.9	0.78	0.257	0.9	0.695	–0.324
CA19.9	0.419	0.9	0.749	0.543	0.9	0.825	0.124
MUC5AC, IL6	0.355	0.9	0.727	0.343	0.9	0.736	–0.012
MUC5AC, CA19.9	0.355	0.9	0.705	0.257	0.9	0.741	–0.098
CA19.9, CRP	0.355	0.9	0.6	0.257	0.9	0.716	–0.098
MUC5AC	0.323	0.9	0.701	0.229	0.9	0.743	–0.094
MUC5AC, TBIL	0.323	0.9	0.699	0.314	0.9	0.74	–0.009
MUC5AC, CRP	0.29	0.9	0.71	0.371	0.9	0.783	0.081
LRG1, CA19.9	0.258	0.9	0.626	0.543	0.9	0.821	0.285
LRG1, CRP	0.194	0.9	0.599	0.343	0.9	0.668	0.149
TBIL, GGT	0.129	0.9	0.542	0.143	0.9	0.683	0.014
TBIL	0.129	0.9	0.537	0.086	0.9	0.668	–0.043
TBIL, CRP	0.097	0.9	0.534	0.314	0.9	0.674	0.217
CRP	0.032	0.9	0.47	0.314	0.9	0.679	0.282

A selection of 48 of the 105 models generated (up to 3 markers) are shown to highlight the effect of TBIL on performance.

Supplementary Table 2: Biomarker levels in pre-diagnosis samples and cancer-free controls

Marker	Biliary tract cancer (*n* = 89)	Control (*n* = 91)	*P* value
TBIL (μmol/L)	4.6 (1.9–20.9)	4.7 (1.7–21.8)	0.916
CRP (mg/L)	3.1 (0.6–71.9)	1.9 (0.6–54.0)	0.0007
ALP (U/L)	83 (29–844)	73 (29–151)	0.006
GGT (U/L)	17 (0.6–2566)	12 (0.6–234)	0.039
CA19–9 (U/mL)	12.2 (1.0–1283)	8.4 (1.0–73.3)	0.002
CA19–9 > 37 U/mL (%)	15/89 (17%)	6/91 (7%)	0.038*
LRG1 (μg/mL)	139.2 (7.56–546.9)	62.78 0.45–836.4)	0.362
IL6 (ng/mL)	16.78 (1.0–1600)	15.37 (1.0–1600)	0.240
PKM2 (ng/mL)	2.46 (0.66–43.03)	2.27 (0.32–7.53)	0.628

Median values (range) are given. *P* values determined using the Mann Whitney *U* test or Fisher's exact test*.
